# EGFP-EGF1-conjugated poly (lactic-co-glycolic acid) nanoparticles as a carrier for the delivery of CCR2− shRNA to atherosclerotic macrophage in vitro

**DOI:** 10.1038/s41598-020-76416-4

**Published:** 2020-11-12

**Authors:** Zhilin Wu, Chen Chen, Jiajia Luo, Jacques R. J. Davis, Bo Zhang, Liang Tang, Wei Shi, Danying Liao

**Affiliations:** 1 Anesthesiology Department, Union Hospital, Tongji Medical College, Huazhong University of Science and Technology, Wuhan, 430022 People’s Republic of China; 2 Haematology Department, Union Hospital, Tongji Medical College, Huazhong University of Science and Technology, Wuhan, 430022 People’s Republic of China

**Keywords:** Drug delivery, Diseases, Atherosclerosis

## Abstract

Reducing macrophage recruitment by silencing chemokine (C–C motif) receptor 2 (CCR2) expression is a promising therapeutic approach against atherosclerosis. However the transfection of macrophages with siRNA is often technically challenging. EGFP-EGF1-conjugated poly (lactic-co-glycolic acid) (PLGA) nanoparticles (ENPs) have a specific affinity to tissue factor (TF). In this study, the feasibility of ENPs as a carrier for target delivery of CCR2-shRNA to atherosclerotic cellular models of macrophages was investigated. Coumarin-6 loaded ENPs were synthesized using a double-emulsion method. Fluorescence microscopy and flow cytometry assay were taken to examine the uptake of Coumarin-6 loaded ENPs in the cellular model. Then a sequence of shRNA specific to CCR2 mRNA was constructed and encapsulated into ENPs. Target delivery of CCR2-shRNA to atherosclerotic cellular models of macrophages in vitro were evaluated. Results showed more uptake of ENPs by the cellular model than common PLGA nanoparticles. CCR2-shRNA loaded ENPs effectively silenced CCR2 gene in the atherosclerotic macrophages and exhibited a favorable cytotoxic profile to cultured cells. With their low cytotoxicity and efficient drug delivery, ENP could be a useful carrier for target delivery of CCR2-shRNA to inflammatory monocytes/macrophages for the therapy against atherosclerosis.

## Introduction

Atherosclerosis is a progressive disease characterized by the accumulation of lipids and fibrous elements in the large and medium-size arteries. It is at the core of cardiovascular disease leading to myocardial infarctions, stroke, and peripheral vascular disease encountered in most human populations^1^.

Elevated level of LDL (low density lipoprotein), homocysteine, reduced level of HDL (high density lipoprotein), metabolic syndromes, systemic inflammation, hypertension, smoking, high fat diets and lack of exercise are risk factors of atherosclerosis^2^. To decrease clinical events and mortality from atherosclerosis, healthier life styles such as smoking cessation, low-fat, high-fiber vegetarian diet and moderated aerobic exercise are advocated^3^. More effective medicines against hyperlipidaemia are developed^4,5^. However, atherosclerosis remains the underlying cause of about 50% of all deaths in westernized societies and accounts for more deaths than any other disease in the whole world^5,6^.

Patients with well controlled hyperlipidaemia and hypertension have a residual risk for ischemic events, which might be ascribed to inflammation^7^. Monocytes/Macrophages are central effectors of innate immunity and play a key role in atherosclerotic vessel related inflammation^8^. Atherosclerosis starts with the recruitment of inflammatory monocytes on the activated endothelial cells. In response to inflammatory stimuli, these monocytes migrate through the impaired endothelium and further proliferate within the intimal layer, sustaining and amplifying the local inflammatory process by releasing inflammatory factors, which mediate the involved immune cells activation, smooth muscle cells migration and proliferation, extracellular matrix production, further fuel the progression of atherosclerotic lesions. The migration and accumulation of monocyte/macrophage is continuous and proportional to the disease progression^9^.

The role of CCR2 in the monocyte recruiting and migration from bone marrow to peripheral sites of inflammation has been firmly established in the literature^10^. Monocytes do not migrate in response to MCP-1 in CCR2 knockout mice. These cells are less capable of adhering to the endothelium, suggesting the importance of CCR2 in the strong inter-reaction between monocytes and endothelium^11^. Blocking CCR2 and inhibiting the recruitment of monocytes to atherosclerotic lesions is one promising strategy to ameliorate the progression of atherosclerosis and stabilize plaques^12^.

With the development of nanotechnology, several nano-therapies were devised for delivery of specific siRNA or antagonist targeting CCR2 in monocytes/macrophages, with an aim to manipulate their recruitment, migration^13,14^ proliferation^15,16^ and differentiation in atherosclerosis^17^. However, these attempts have largely failed due to off-target effects and suppression of host defense. To improve drug delivery efficacy and decrease side effects, specific monocytes/macrophages targeting nanocarrier is urgently needed.

In our previous work, we have developed an EGFP-EGF1 nanoparticle (ENP), a targeting drug delivery system which has a specific affinity to TF protein on cell surface^18^. It has been proved that ENP’s can mediate target delivery of TF-siRNA to injured brain micro-vascular endothelial cells (BMECs) for effective TF gene silencing in rats^19^. We also found that ENPs exhibited specific binding to atherosclerotic cellular model of vessel smooth muscle cells and plaques in an animal model^20^. We hypothesize that ENPs could also effectively adhere to atherosclerotic macrophages via binding to TF protein on cell surface. Thus they could work as effective pharmaceutical carriers to manipulate the involvement of monocytes/macrophages in the pathogenesis of this inflammatory disease and make the prevention and therapy of the disease more targeting. In this research, a cellular model of atherosclerotic macrophages was established and uptake test of ENPs was carried out. Then a specific CCR2-shRNA was constructed and loaded into ENPs. Their effect on the CCR2 gene silencing was investigated.

## Materials and methods

### Materials and cells

The E. coli strain BL21 (DE3) and plasmid pET-28a-EGFPEGF1 were maintained in our laboratory. Poly-(d, l lactic-coglycolic acid) (PLGA, 50:50, inherent viscosity of 0.89, MW, 100 kDa) was purchased from Absorbable Polymers (USA). Methoxy-poly-(ethylene glycol) (M-PEG, MW 3000 Da) was purchased from the NOF Co. (lot no.14530, Japan) and Maleimide-PEG (Mal-PEG, MW 3400 Da) was purchased from Nektar Co. (lot no.PT-08D-16, USA). Rabbit polyclonal antibody against rat TF antibody was purchased from Santa Cruz Biotechnology Co. (USA). Dulbecco's Modified Eagle Medium (DMEM) (high glucose), RPMI 1640 Medium and fetal bovine serum (FBS) were purchased from Thermo Fisher Scientific Technologies Co. (USA). OxLDL (oxidized LDL) was purchased from Beijing Yiyuan Bio Technology Co. (China). Raw264.7 cells (Chinese Academy of Medical Sciences and Peking Union Medical College, Beijing, China) were cultured in RPMI 1640 (high glucose) which consisted of 10% FBS and antibiotics (including 100 mg/ml of penicillin and 100 mg/ml of streptomycin) at 37 °C in a humidified atmosphere with 5% CO_2_. The following forward and reverse oligonucleotides were used for construction of CCR2-shRNA plasmid as reported in literature^21^ (Primers-F: 5-AGCCTCGAGATGGAAGACAATAATATGTT-3; Primers-R: 5-ATAAAGCTTTTACAACCCAACCGAGACCTCT-3).

### Characterization and loading efficiency measurement of nanoparticles

CCR2-shRNA loaded PLGA nanoparticles, Coumarin-6 loaded EGFP-EGF1- PLGA nanoparticles and CCR2-shRNA loaded EGFP-EGF1-PLGA nanoparticles were prepared using a water in oil in water (w/o/w) double emulsion solvent evaporation method as previously described^22,23^. The characterization and loading efficiency of nanoparticles were determined as before^20^. Drug loading capacity (DLC) and drug encapsulation efficiency (DEE) were calculated according to the following equations:

$$ \begin{aligned} {\text{DLC}} & = {\text{the}}\,{\text{weight}}\,{\text{of}}\,{\text{shRNA}}\,{\text{in}}\,{\text{the}}\,{\text{nanoparticles}}/{\text{the}}\,{\text{weight}}\,{\text{of}}\,{\text{the}}\,{\text{nanoparticles}} \\ {\text{EE}}\% & = \left( {{\text{actual}}\,{\text{shRNA}}\,{\text{loading}}/{\text{theoretical}}\,{\text{shRNA}}\,{\text{weight}}} \right) \times 100\% \\ \end{aligned} $$

### Cellular model establishment and mRNA measurement

Raw264.7 cells were seeded at a density of 1 × 10^5^ per well in 6-well, incubated for 12 h. Then the medium was changed for the serum-free one. Since the process of oxLDL induced macrophage foaming is particularly important for atherosclerosis progression^24^, a concentration of 50 μg/ml oxLDL^25,26^ was used for Raw264.7 cells induction for the establishment of atherosclerotic cellular model of macrophage in this in vitro study. Treated cells were collected for real time PCR to measure TF mRNA and CCR2 mRNA levers at 0, 0.5, 1, 2, 4, 8, 10, 12, 24 h, respectively. The following primers were used for real-time PCR: TF Primer-F (5′–3′) GCACCGAGCAATGGAAGAG Primer-R (5′–3′) CAGAGATATGGACAGGAGGATGAT.

CCR2 Primer-F (5′–3′) CGGCATACTATCAACATCTCATTC: Primer-R (5′–3′) CAAGGCTCACCATCATCGTA.

GAPDH Primer-F (5′–3′) ATGGTGGTGAAGACGCCAGTA GAPDH: Primer-R (5′–3′) GGCACAGTCAAGGCTGAGAATG.

### Cellular uptake study

Coumarin-6 loaded ENPs or NPs were added and the mixture was incubated for 30 min at 37 °C. The cells were washed 2 times with PBS (0.01 M, pH 7.4) and immobilized with 4% paraformaldehyde for 20 min at room temperature. Then the fluorescence intensity of the cells was detected by flow cytometry and fluorescence microscopy respectively. Untreated cells were incubated with the empty nanoparticles to serve as a control.

### In vitro transfection experiments

The Raw264.7 cells were seeded at a density of 1 × 10^5^ per well in 6-well plates and grew to reach 70–80% confluence. The Raw264.7 cells were treated with oxLDL (50 μg/ml) for 1 h. The fresh serum-free RPMI 1640 containing different nanoparticle formulations, including NPs, ENPs, CCR2-shRNA loaded NPs, and CCR2-shRNA loaded ENPs were incubated at 37 °C in a 5% CO_2_ humidified atmosphere for 6 h. A negative control was prepared with PBS alone. After the treatment, the cells were washed 2 times with PBS, trypsinized by 0.25% trypsinase and collected. The CCR2 and TF expressions were determined by real time PCR and western blot.

### Cytotoxicity assay

The Raw264.7 cells were seeded in 24-well plates at a density of 5 × 10^4^ cells per well in M131 and incubated for 24 h. The cells were then exposed to PBS, CCR2-shRNA, EGF1, LTX, PLGA-NP, or ENP. There were four wells for each mixture. Twenty four hours following exposure, 40 μl of CCK-8 (Dojindo, Japan) was added to each well, and the mixtures were incubated for 4 h. The absorbance (A) was measured at 450 nm with a microplate reader (BioTek, USA). The cell viabilities were normalized using blank cells.

### Statistical analysis

Statistical analysis was made using GraphPad Prism 5.0. Data was shown as mean ± SD. Statistical comparisons between two groups were evaluated by Student’s t-test. Comparisons between more than two groups were made by one-way analysis of Variance (ANOVA). Differences were considered statistically significant at *p* ≤ 0.05 for all analyses.

## Results

### Characteristics and loading efficiency of nanoparticles

The PLGA NPs were prepared using double-emulsion solvent evaporation method and conjugated with the EGFP-EGF1 fusion protein. Then coumarin-6 or CCR2-shRNA was loaded into the NPs/ENPs. Each sample was analyzed three times. DLS showed the mean size of coumarin-6 and CCR2-shRNA loaded NPs/ENPs was about 100 nm. Their zeta potential was around -10 mV (Table 1). DEE of ENP and NP were 78.35 ± 0.34% and 79.37 ± 0.13%. DLC of ENP and NP were 1.33 ± 0.02 μg/mg and 1.34 ± 0.03 μg/mg. Results showed that shRNA loaded ENPs and NPs were appropriate for the following experiments (Table 2).

**Table 1:** Particle size and zeta potential of nanoparticles.

Nanoparticles	Mean size (nm)	Zeta potential (mV)
Coumarin-6-ENP	105.06 ± 2.32	− 10.61 ± 1.01
Coumarin-6-NP	98.86 ± 1.42	− 10.31 ± 2.18
CCR2-shRNA-ENP	106.08 ± 1.33	− 10.35 ± 3.09
CCR2-shRNA-NP	99.25 ± 2.52	− 10.53 ± 1.73

Measured in double-distilled water (n = 3), mean ± SD.

**Table 2:** DEE and DLC of shRNA-loaded NPs and ENPs.

Nanoparticles	CCR2-shRNA-ENPs	CCR2-shRNA-NPs
Drug encapsulation efficiency (%)	78.35 ± 0.34	79.37 ± 0.13
Drug loading capability (μg/mg)	1.33 ± 0.02	1.34 ± 0.03

Measured in double-distilled water (n = 3), mean ± SD.

### CCR2 and TF mRNA expression increased in oxLDL stimulated Raw264.7

**Figure 1:**
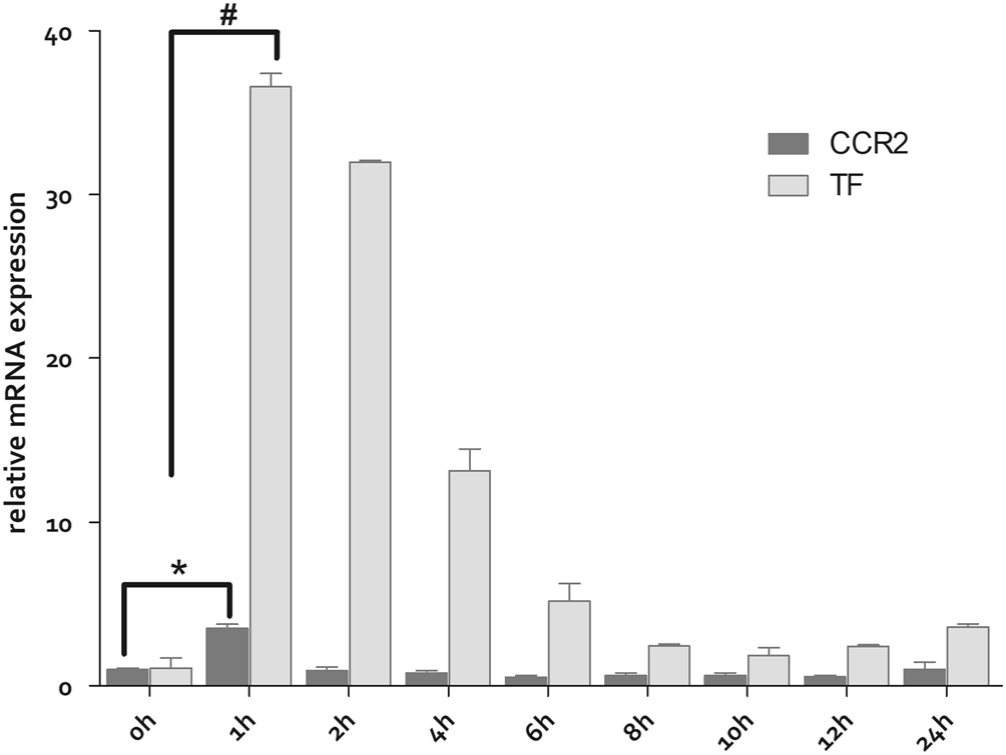
The TF and CCR2 mRNA expression were determined by Q-PCR. The experiments were performed in triplicate (mean ± SD). The mRNA levels from the differently treated Raw264.7 cells were normalized to the GAPDH mRNA level. The relative fold of the TF and CCR2 mRNA levels from the treated Raw 264.7 was normalized to the negative control from the normal Raw264.7 cells. *P < 0.01, ^#^*P* < 0.01.

It could be seen that TF and CCR2 mRNA increased simultaneously in Raw264.7 cells with the stimulation of 50 μg/ml oxLDL^24–26^. And their expression reached its peak level after 1 h of exposure. Then after 2 h of exposure, CCR2 decreased and returned to normal level. TF also decreased, but still maintained at a relative high level (Fig. 1). It suggested that longer time exposure of oxLDL might impair the expression of CCR2 and TF from its toxic effect. Therefore, cellular model stimulated with oxLDL for 1 h was selected for subsequent nanoparticle uptake test and nanoparticle delivered mRNA interference experiments.

### Effective uptake of ENP determined by fluorescence microscopy and flow cytometry examination

**Figure 2:**
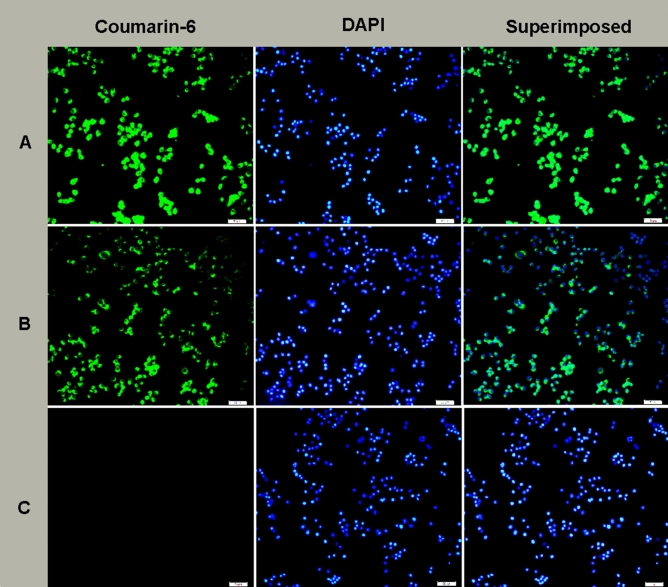
Uptake of coumarin-6 loaded nanoparticles in oxLDL stimulated Raw 264.7. (**A**) Raw 264.7 incubated with 6-coumarin labeled ENPs at 37 °C for 0.5 h. (**B**) Raw 264.7 incubated with 6-coumarin labeled NPs at 37 °C for 0.5 h. (**C**) Raw 264.7 incubated alone with 0.01 M PBS (pH 7.4) at 37 °C for 0.5 h. The bars are 100 μm.

**Figure 3:**
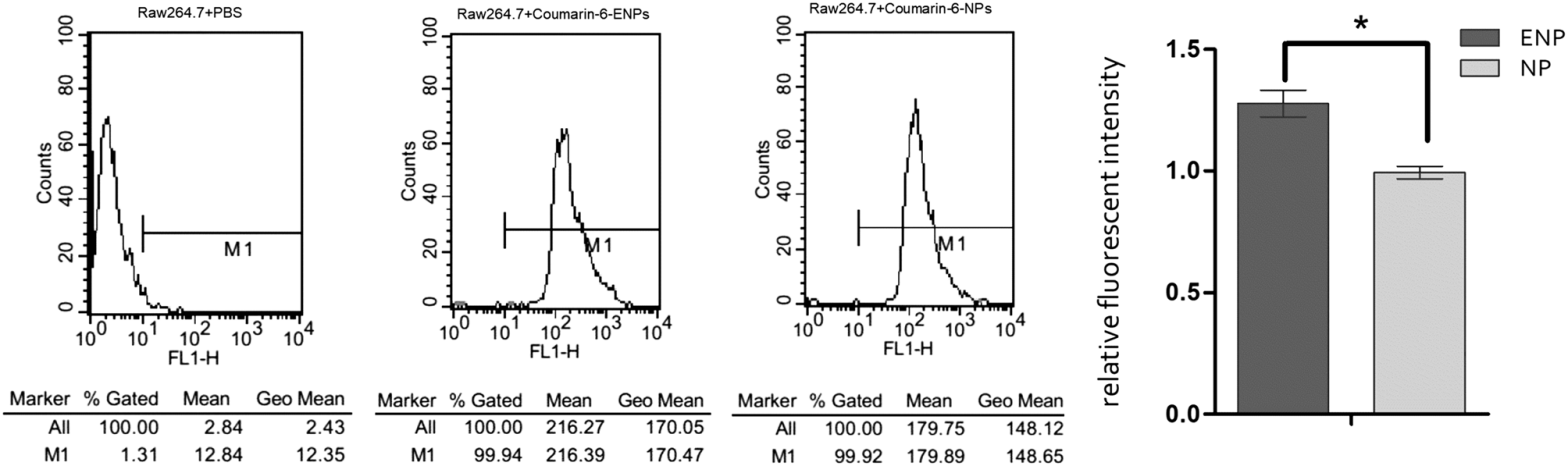
Cell fluorescence intensity of Raw264.7 cells. The experiment was repeated three times to calculate the average fluorescence intensity of each group, and the histogram was obtained based on NP group (mean ± SD). *P < 0.01.

Raw264.7 cells were exposed to oxLDL (50 μg/ml) for 1 h. Then the cellular model was incubated with coumarin-6 loaded ENPs and coumarin-6 loaded NPs. A much stronger fluorescence was observed in the ENP group than in the NP group, indicating that ENPs could be effectively absorbed by the cellular model (Fig. 2). Flow cytometry was taken for the quantitative analysis of the fluorescence intensity. As shown in Fig. 3, fluorescence intensity was significantly higher in the ENP group than in the NP group. The results were consistent with those of the fluorescence microscopy.

### Interference on CCR2 mRNA expression with CCR2-shRNA loaded ENPs

**Figure 4:**
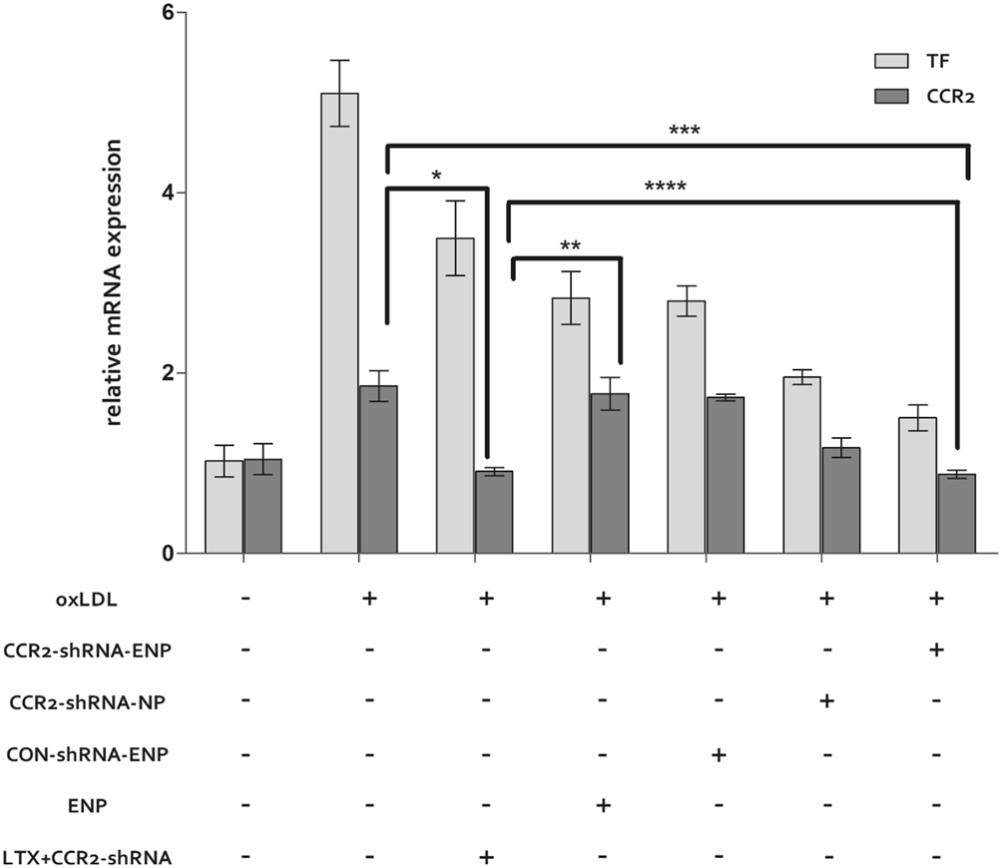
CCR2 and TF mRNA expression in different transfection agent treated cells. The TF and CCR2 mRNA expression were determined by Q-PCR. The experiments were performed in triplicate (mean ± SD). The mRNA levels from the different nanocarrier treated cells were normalized to the GAPDH mRNA level. The relative fold of the TF and CCR2 mRNA levels from the nanocarrier treated Raw 264.7 was normalized to the negative control from the normal cells. *P < 0.05, **P < 0.05, ***P < 0.05, ****P > 0.05.

After the stimulation with oxLDL (50 μg/ml) for 1 h, the Raw264.7 cells were transfected with CCR2-shRNA loaded nanoparticles. Total RNA and protein were extracted after 24 h and 48 h respectively. As shown in Fig. 4, CCR2 and TF mRNA increased sharply under the stimulation of oxLDL and maintained at a high level even after 24 h. However, the mRNA induction with oxLDL was attenuated significantly when treated with CCR2-shRNA-ENP or CCR2-shRNA-LTX. Both could effectively decrease CCR2 and TF mRNA levels. The CCR2 mRNA levels in CCR2-shRNA-NP treated cells also decreased, but were less efficient than CCR2-shRNA-LTX and CCR2-shRNA-ENP. Empty ENP could decrease TF expression in the cellular model, but had no effect on CCR2 mRNA expression.

### Inhibition of CCR2 protein expression with CCR2-shRNA loaded ENPs

**Figure 5:**
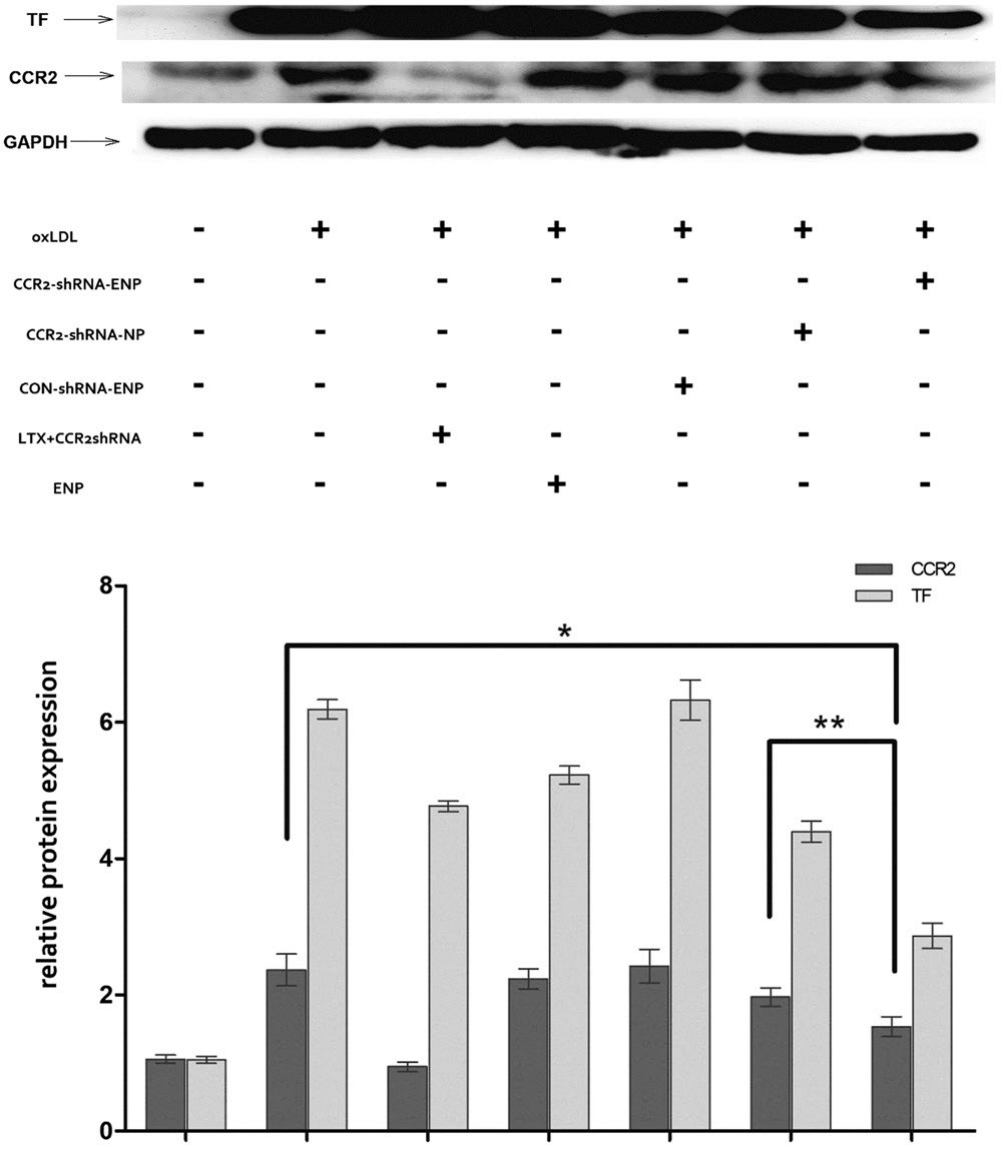
CCR2 and TF protein expressions in different transfection agent treated cells. The TF and CCR2 protein expressions were determined by western-blot. The experiments were performed in triplicate (mean ± SD). The protein levels from the different transfection agent treated cells were normalized to the GAPDH protein level. The relative fold of the TF and CCR2 protein from the treated Raw 264.7 was normalized to the negative control from the normal cells. *P < 0.05, **P < 0.05.

As shown in Fig. 5, CCR2 and TF protein expressions in the cellular model maintained at a high level with oxLDL stimulation. Their expressions were significantly inhibited by CCR2-shRNA loaded LTX exhibited the strongest effect of CCR2 protein expressions inhibition. Though not as powerful as CCR2-shRNA loaded LTX, both CCR2-shRNA-ENPs and CCR2-shRNA-NPs could also significantly inhibit the expression of CCR2 protein expression. CCR2-shRNA-ENPs worked more efficiently for CCR2 protein expression inhibition than CCR2-shRNA-NPs.

### Favourable cytotoxicity profile of CCR2-shRNA loaded ENPs determined by CCK-8 assay

**Figure 6:**
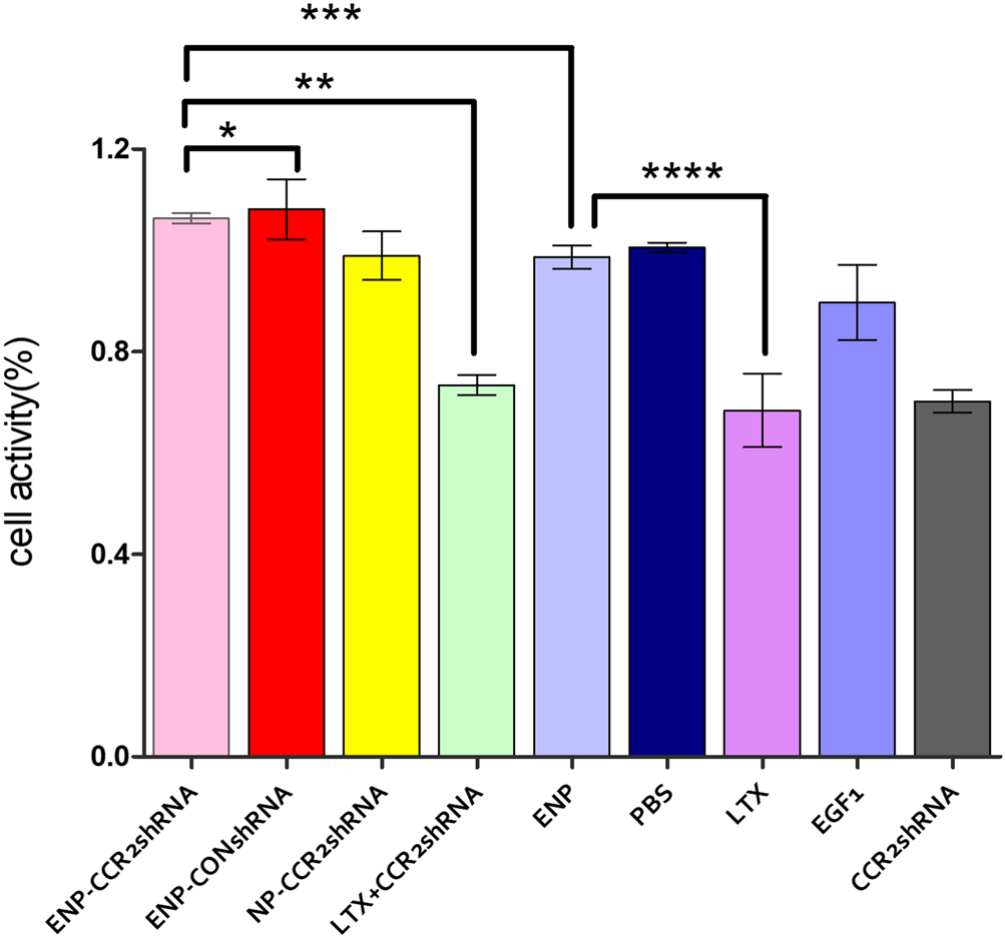
The activity of different nanocariers and solutions treated Raw 264.7 cells were normalized to activity of the normal cells. The assays were performed in triplicate (mean ± SD). *P *>* 0.05, **P < 0.05, ***P > 0.05, ****P *<* 0.05.

After incubation with different kinds of nano-carriers and solutions for 24 h, the absorption values of each group were determined by CCK-8 assay and the cell activity of each group was calculated with four compound holes per group. As shown in Fig. 6, CCR2-shRNA loaded ENPs and ENPs exhibited a same favorable cytotoxic profile as **N-shRNA loaded ENPs**. There was nearly no toxic effect on cell activity. CCR2-shRNA loaded LTX had an adverse effect on cell activity. LTX itself revealed a significant toxic effect on cell activity. Cell activity also decreased with 0.1 μg CCR2-shRNA alone.

## Discussion

Chronic inflammation is a central pathogenic mechanism of atherosclerotic induction and progression. Vascular inflammation is associated with accelerated onset of late atherosclerosis complications. Systemic administration of nonspecific agents as mathotrexate, colchicine, aspirin and some specific modulators to key pro-inflammatory signaling pathways were tried for anti-inflammation therapy^27^. Despite the evidence of the efficacy of the present treatments, the complex and poorly understood underlying mechanisms of multiple regulatory and compensatory mechanisms evolved in atherosclerotic inflammation have prevented them from reaching their full potential^28^. Anti-inflammatory treatment of atherosclerosis remains challenging. As evidenced by numerous studies that monocytes’ accumulation and subsequent chronic inflammation is involved in atherogenesis from lesion initiation, foam cell formation, and pooling of apoptotic cells that affect plaque size and vulnerability, macrophages have continuously been the subject in the research for prevention and treatment of atherosclerosis^29^.

In our previous work, we synthesized a stable EGFP-EGF1-PLGA nanoparticle. With EGFP-EGF1 fusion protein, which has a specific affinity to tissue factor protein, ENPs could be effectively absorbed by injured BMECs, neuroglial cells and glioma cells and work as a carrier for targeting drug delivery. The nanoparticle has been proved to be an effective carrier for anti-glioma delivery of paclitaxel (PTX) by targeting both neovascular and glioma cells^23,30^. High level of TF protein were synthesized in circulating monocytes and lipid laden macrophages in plaques^31^. The selective combination of ENP with TF protein allows us to consider its utility in targeting therapy of monocytes/macrophages in atherosclerosis.

In this study, we established an atherosclerotic cellular model of macrophage with cell line Raw264.7. By inducing with oxLDL for 1 h, TF mRNA and CCR2 mRNA increased significantly. Tissue factor (TF) is an initiator of the extrinsic coagulation cascade^32^, and it is expressed in peripheral blood monocytes and macrophages in atherosclerotic plaque. It indicated that the cellular model had acquired pro-coagulatory and enhanced migratory capability and exhibited similar character of circulating monocytes in atherosclerosis.

With double emulsion method, Coumarin-6 loaded ENPs were synthesized and exposed to atherosclerotic macrophages. Fluorescent microscopy examination was performed for the uptake test of ENPs in the cellular model. Significantly stronger fluorescence could be observed in ENPs’ group than in NPs’ group. The results suggested that with affinity of fusion protein EGFP-EGF1 to TF protein on cell surface, ENPs could be more efficiently absorbed by the cellular model of macrophages than NPs. The high efficiency of uptake indicates ENPs could be an ideal carrier for target drug delivery to atherosclerotic monocytes/macrophages.

In patients with hyperlipidemia, circulating monocyte CCR2 levels were significantly correlated with plasma LDL and cholesterol levels and were positively associated with monocyte inter-cellular lipid accumulation^33^. In our experiment, the level of CCR2 in macrophages increased dramatically under the stimulation of oxLDL, which also proved the close relationship between CCR2 and hyperlipideamia in atherosclerosis. Monocyte CCR2 expression is associated with arterial wall inflammation in patients at increased cardiovascular risk and it is independent of traditional cardiovascular risk factors and statin use^34^. The mobilization and recruitment of monocytes/macrophages to the early atherosclerotic plaque is mediated by CCR2-CCL2 interaction^35^. CCR2 and ApoE double knockout mice exhibited a threefold reduction of atherosclerotic lesions in mean aortic area compared to apoE-deficient control mice^11^. Many efforts have been made to develop biologic small molecule antagonists for CCR2 blockage^36,37^. However the number of compounds that have shown in vivo efficacy in inhibiting atherosclerosis is very limited^38^. Small interference RNA (siRNA) technology brings promise to attenuate production of specific target proteins in vivo by degradation of mRNA^39^. With an in vivo application of lipid nanoparticles containing siRNA against CCR2, a reduction in the number of classical monocytes at the sites of inflammation and therapeutic effects have been achieved in acute and chronic inflammatory disease models^40–42^.

We constructed a specific CCR2 interference shRNA and loaded it into ENPs. The gene silencing effect of CCR2-shRNA ENPs to atherosclerotic macrophages was investigated. Results showed that CCR2-shRNA encapsulated ENPs could more efficiently inhibit CCR2 expression than NPs. PLGA is one of the most widely used biodegradable polymeric nanoparticle that is approved by FDA^43,44^. Fusion protein EGFP-EGF1 is synthesized based on the TF gene in the EGF1 region and has a specific affinity to TF. The conjugation of PLGA with EGFP-EGF1 fusion protein makes the nanoparticles more targeting to macrophages with TF expressions. Changes of CCR2 in monocytes correlated with their migratory capacity. The results suggest that CCR2 shRNA encapsulated ENP’s might be used to reduce the migration of macrophage in atherosclerosis by down regulation of CCR2 level. Interestingly, there might be some coupling mechanism between the TF and CCR2 expressions. The blockade of CCR2 expression by different naonocarriers loaded CCR2 shRNA also down-regulated the expression of TF, especially with CCR2-shRNA encapsulated ENPs. However, ENP exhibited the highest shRNA delivery efficiency among all carriers, which was probably ascribed to the selective combination of ENP with TF, even in cells with low TF level.

Cationic liposomes, PLGA, and lipid species are commonly used bio-active agents for delivery of nano-carriers for precision targeted therapeutics on monocytes/macrophages in inflammatory diseases^45,46^. However macrophages are refractory to these non-viral approaches for cytotoxicity of the nanoparticles or damage of interfering RNA by their own degenerative enzymes^47^. To be clinically useful, the nano-carrier itself should be non-toxic. CCK8 assay was taken to observe ENPs’ cytotoxicity. It showed that there was nearly no toxic effect of ENPs on cell viability. Only a mild toxic effect was found in shRNA loaded ENPs. While liposome LTX had an obvious cytotoxicity to macrophages. Thus the higher efficiency of CCR2 inhibition by LTX than ENP might partly be ascribed to their toxic effect on cell viability. With favourable bio-compatibility and cell selectivity, it can be inferred that ENP is more suitable for interference RNA delivery to atherosclerotic macrophages than LTX.

While our study highlights the advantage of ENP for efficient CCR2 shRNA delivery to atherosclerotic macrophages, there are several limitations in our research. First, this is an experiment in vitro, with the difference between a cellular model and an animal model, the effect of target CCR2 shRNA delivery to circulating monocyte in-vivo is urgently required to be studied. Second, as the cellular model in our experiment is mimicking mature foam cells, the effect of CCR2-shRNA ENPs to an early stage atherosclerotic monocytes is to be studied, which might be important for the prevention and early therapy of atherosclerosis. Third, ENPs may not be completely specific to atherosclerotic monocytes. Other cell types such as neutrophils and lymphocytes that are involved in the formation of atherosclerotic lesions do also express a certain amount of TF proteins. The effect of CCR2- shRNA loaded ENPs in these cell types needs to be studied.

In summary, our data demonstrate that CCR2-shRNA loaded ENP could be effectively up taken by atherosclerotic cellular model of macrophages and target-silence their CCR2 mRNA expressions, which suggests the nanoparticle’s potential application for therapy of atherosclerosis. With its bio-compatibility and efficient drug delivery to macrophage, it can also work as a new platform for RNAi based therapies in other macrophage associated inflammatory diseases.

## Supplementary information

Supplementary information.
