# Supplementary material for: EGFP-EGF1-conjugated poly (lactic-co-glycolic acid) nanoparticles as a carrier for the delivery of CCR2− shRNA to atherosclerotic macrophage in vitro
Source: Sci Rep. 2020 Nov 12;10:19636. doi: 10.1038/s41598-020-76416-4 (PMC7661524; doi:10.1038/s41598-020-76416-4)
Supplement: Supplementary file 1 — Supplementary information. [file 41598_2020_76416_MOESM1_ESM.pdf]

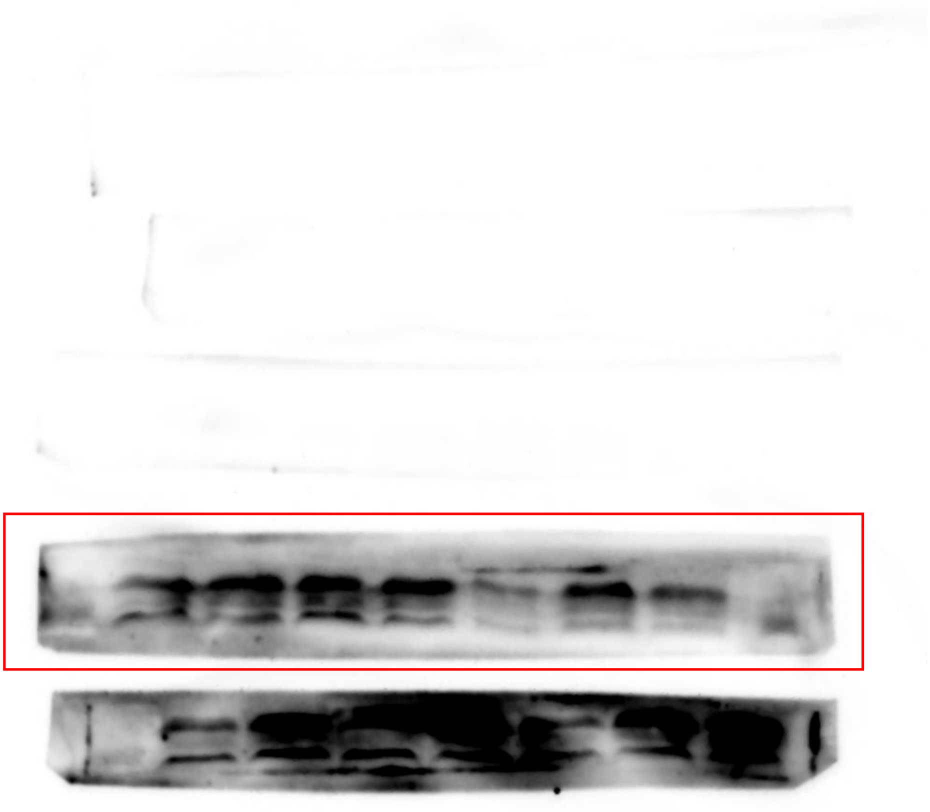

CCR2

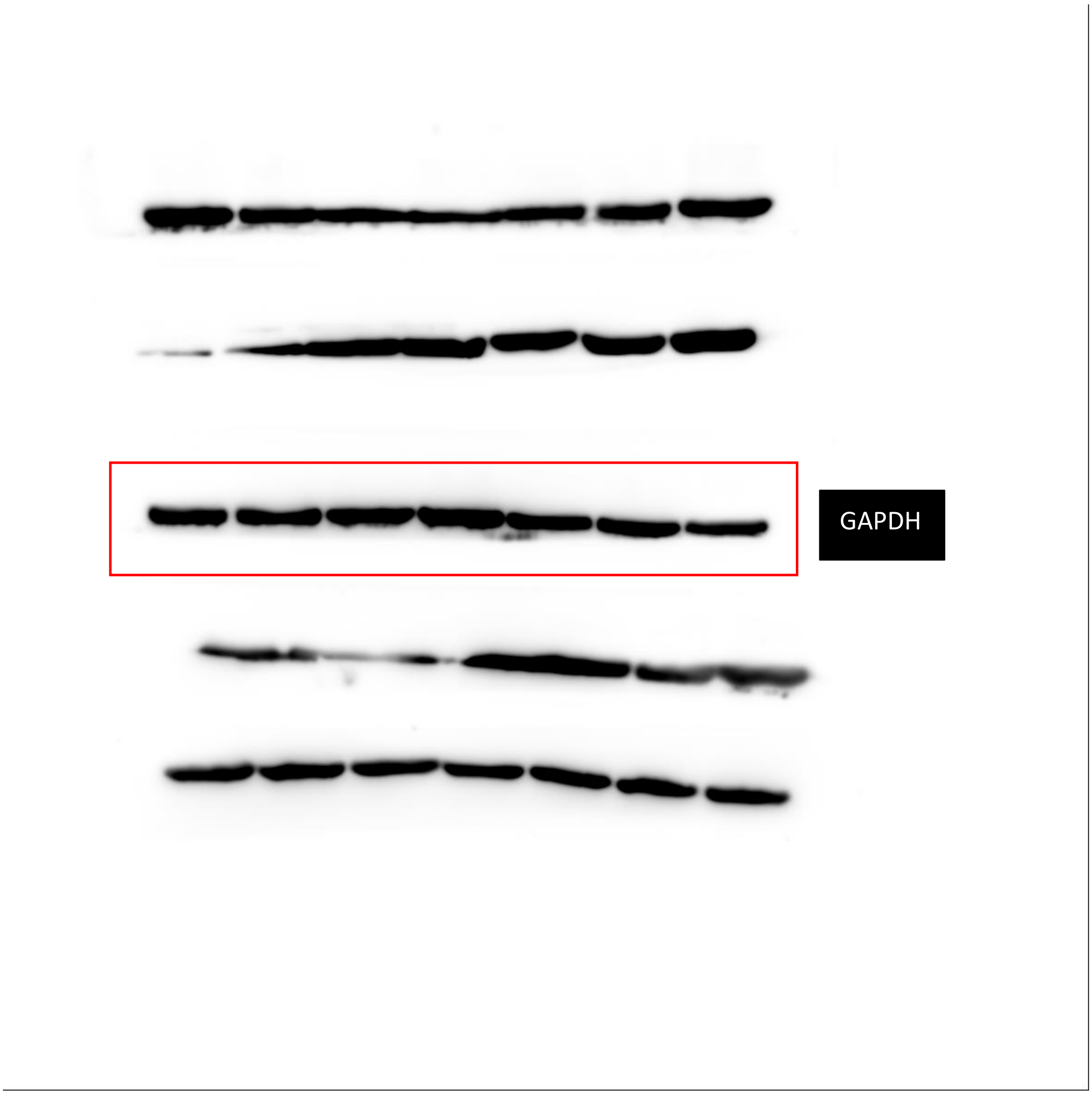
GAPDH

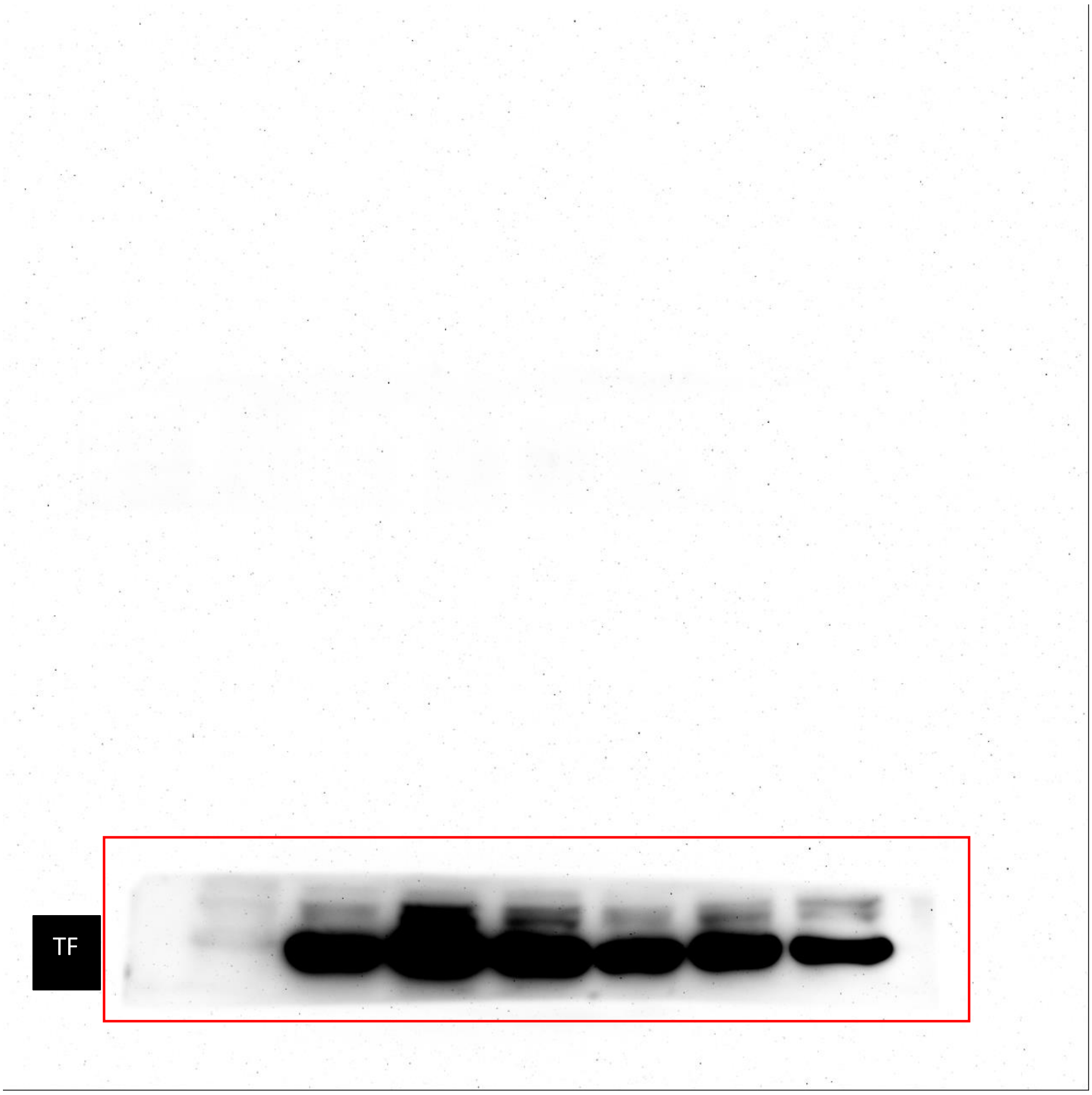

The above photos were original western-blot for CCR2, Tissue factor and GAPDH protein expressions. Heterozona coexists in the blot and was chopped to make the figures more beautiful.
